# Topical estrogen, testosterone, and vaginal dilator in the prevention of vaginal stenosis after radiotherapy in women with cervical cancer: a randomized clinical trial

**DOI:** 10.1186/s12885-021-08274-w

**Published:** 2021-06-10

**Authors:** Jumara Martins, Ana Francisca Vaz, Regina Celia Grion, Lúcia Costa-Paiva, Luiz Francisco Baccaro

**Affiliations:** 1 Radiotherapy Section, Woman’s Hospital - Caism/Unicamp, Campinas, SP Brazil; 2 Department of Gynecology, Faculty of Medical Sciences, State University of Campinas - UNICAMP, Rua Alexander Fleming, 101, Cidade Universitária Zeferino Vaz, Campinas, SP 13083-881 Brazil

**Keywords:** Uterine cervical neoplasm, Brachytherapy, Adverse effects, Radiotherapy

## Abstract

**Background:**

We aimed to evaluate the effects of different therapeutic options to prevent the evolution of vaginal stenosis after pelvic radiotherapy in women with cervical cancer.

**Methods:**

open-label randomized clinical trial of 195 women, stage I-IIIB, aged 18–75 years, using topical estrogen (66), topical testosterone (34), water-based intimate lubricant gel (66), and vaginal dilators (29) to assess the incidence and severity of vaginal stenosis after radiotherapy at UNICAMP-Brazil, from January/2013 to May/2018. The main outcome measure was vaginal stenosis assessed using the Common Terminology Criteria for Adverse Events (CTCAE) scale and percental changes in vaginal volume. The women were evaluated at four different times: shortly after the end of radiotherapy, and four, eight, and 12 months after the beginning of the intervention. Statistical analysis was carried out using Symmetry test, Kruskal-Wallis test and multiple regression.

**Results:**

the mean age of women was 46.78 (±13.01) years, 61,03% were premenopausal and 73,84% had stage IIB-IIIB tumors. The mean reduction in vaginal volume in the total group was 25.47%, with similar worsening in the four treatment groups with no statistical difference throughout the intervention period. There was worsening of vaginal stenosis evaluated by CTCAE scale after 1 year in all groups (*p* < 0.01), except for the users of vaginal dilator (*p* = 0.37).

**Conclusions:**

there was a reduction in vaginal volume in all treatment groups analyzed, with no significant difference between them. However, women who used vaginal dilators had a lower frequency and severity of vaginal stenosis assessed by the CTCAE scale after one year of treatment.

**Trial registration:**

Brazilian Registry of Clinical Trials, RBR-23w5fv. Registered 10 January 2017 - Retrospectively registered.

**Supplementary Information:**

The online version contains supplementary material available at 10.1186/s12885-021-08274-w.

## Introduction

Cervical cancer is the fourth commonest cancer in women worldwide [1]. In Brazil, incidence and mortality rates are intermediate in terms of developing countries, but high compared to those in developed countries with well-structured early-detection programs [1–4]. According to the Sedlis criteria, the initial standard treatment is surgery associated with radiotherapy; and for more advanced cases, the standard therapy comprises concomitant radiotherapy and chemotherapy [5]. The combination of teletherapy and brachytherapy for advanced-stage tumors has a higher complete remission rate, lower recurrence rate, and improved overall disease-free survival compared to exclusive teletherapy and boost in the tumor [6–8].

With increased survival rates of women with cervical cancer, there is an increase in late treatment-related adverse events. Vaginal stenosis, defined as decreased diameter and/or length of the vagina, is one of the most frequent (59–88%) and may be multifactorial in origin [9–12]. Ionizing therapy leads to decreased blood supply, loss of collagen and elasticity, and associated tissue fibrosis, especially in women treated with high doses [10, 13] and associated chemotherapy [14]. In addition, treatment may lead to ovarian failure, with declining serum estrogen concentrations and consequent genitourinary menopause syndrome [15, 16]. Besides resulting in narrowing and shortening of the vaginal canal, symptoms such as dryness, dyspareunia, pruritus, and urinary incontinence may occur [17, 18].

Currently, prevention and treatment of vaginal stenosis is based on the use of a vaginal dilator or topical estrogen [19]. A 2014 systematic review evaluated vaginal dilator use during or immediately after radiotherapy, showing insufficient evidence that regular vaginal dilation prevents the late effects of radiotherapy; furthermore, injury may occur in rare cases [20]. Topical estrogen use has proven effects in treating menopausal genitourinary syndrome [21]. However, its specific use in stenosis after radiotherapy is unclear [22]. Systemic testosterone, successful in the treatment of female sexual dysfunction [23], has been tested using low intravaginal doses in the treatment of genitourinary menopause syndrome with positive preliminary results [24]. A possible increase in the number of vaginal intercourses resulting from improved sexual dysfunction could help prevent stenosis. Besides that, topical testosterone acts on androgen receptors in the vaginal epithelium and may be locally converted to estrogen through the action of the aromatase enzyme [25]. We are not aware of any previous studies on the use of topical testosterone for prevention or treatment of vaginal stenosis after pelvic radiotherapy.

Vaginal stenosis impairs various aspects of the daily life of cervical cancer survivors and possibly hinders early detection of cancer recurrences [9]. To evaluate the effects of different therapeutic options to prevent the evolution of vaginal stenosis after pelvic radiotherapy in women with cervical cancer, we conducted a randomized clinical trial in the Radiotherapy Sector of UNICAMP Women’s Hospital.

## Methods

An open-label randomized clinical trial using topical estrogen, testosterone, water-based intimate lubricant, and vaginal dilators to assess the incidence and severity of vaginal stenosis in women with cervical cancer after radiotherapy was performed at UNICAMP Women’s Hospital - CAISM, from January 2013 to May 2018. The study was approved by the University of Campinas Ethics Committee (number 01301512.0.0000.5404), funded by the São Paulo State Research Support Foundation (FAPESP), process number 2012 / 09215–7, and registered with the Brazilian Registry of Clinical Trials, number NRB-23W5FV. All participants gave informed consent.

### Subject selection

Women with cervical cancer, stage I to IIIB according to the International Federation of Gynecology and Obstetrics [26], aged between 18 and 75 years, who had not undergone hormone therapy in the previous 6 months and who intended undergoing all radiotherapy treatment at the hospital, were invited to participate. Exclusion criteria were previous radiotherapy for cervical cancer, contraindication for estrogen use (recent myocardial infarction, severe arterial hypertension, refractory diabetes mellitus, history of thromboembolism, decompensated liver disease, history of breast cancer, mammary dysplasia with atypical hyperplasia, and a family history of breast cancer), ulcerative colitis, Crohn’s disease, or diarrhea due to intestinal disease.

### Radiotherapy treatment

Women with cervical cancer were treated with external beam radiotherapy (EBRT), brachytherapy or both, with or without concomitant chemotherapy. Women who did not undergo surgery were treated with radiotherapy and chemotherapy (cisplatin at a dose of 40 mg/m^2^ weekly for 6 weeks). EBRT was performed in a Siemens Mevatron 74 linear accelerator (Siemens Medical IMC, Concord, USA), using 10 MV energy, conformal 3D pelvic treatment, whose treatment volume encompassed the tumor and pelvic lymphatic chains. The EBRT dose was 45–50.4 Gy, with a daily fraction of 1.8 Gy, and dose reinforcement of 9–14.4 Gy in cases with compromised parametrium. Brachytherapy was high dose rate, after-loading, with Iridium-192 source, in the Nucletron microSelection Classic brachytherapy device (Nucletron Corporation, Veenendaal, Netherlands) guided by X-ray (2D). Early stage women who had previously undergone surgery were treated with brachytherapy in the vaginal vault with vaginal cylinders. The prescribed dose was 4 Gy calculated at 5 mm from the cylinder surface and 2 cm extension (once a week, four times, total dose 16 Gy). Early stage women who had not previously undergone surgery were treated exclusively with brachytherapy, with probe and ring (dose 7 Gy at Point A, once a week, four times, total dose 28 Gy). Advanced stage women, who had not previously undergone surgery, when treated with brachytherapy used probe and ring (dose 7 Gy at Point A) or probe and cylinder (7 Gy dose at 2 cm from the probe and on the cylinder surface, once a week, four times, total dose of 28 Gy). Geometric Point A corresponds to a point 2 cm above and 2 cm lateral to the intrauterine probe keel, according to the *International Commission on Radiation Units & Measurements 38* (ICRU 38) [27]. We used cylinders with diameters of 30, 35, and 40 mm and rings with sizes 26, 30, and 34 mm. The treatment protocol and dose limits (maximum dose) at the rectum and bladder points followed ICRU 38 [27] and doses were restricted to 500 cGy for the rectum point and 500 cGy for the bladder point (70% of the dose in the point A) for each application of brachytherapy, following the protocol of the Radiotherapy Sector of UNICAMP Women’s Hospital. If the dose at the rectum or bladder point exceeded the limit of 500 cGy, the prescription dose was reduced so that the dose limit for the rectum and bladder points was respected in each application of brachytherapy.

### Intervention

Estrogen, testosterone and lubricating gel were all administered through a graduated applicator. Estrogen and testosterone creams were handled in a specialized pharmacy. Lubricating gel was used as a control group. Women were instructed to apply the product at night when they went to sleep. In the lying position, with the legs flexed and separated, they should introduce the applicator gently and as deeply as possible into the vagina. They should remain lying down for a few minutes after application, so that the product is not expelled immediately after use.

#### Estrogen group

Conjugated estrogen cream (0.625 mg/g, 26 g tube) 1 g vaginally thrice weekly.

#### Testosterone group

Testosterone propionate cream (300 mcg/mL, 25 mL tube) 1 mL vaginally thrice weekly.

#### Lubricant gel group

Water-based lubricating vaginal gel (KY gel®) 3 g vaginally thrice weekly.

#### Vaginal dilator group

An intravaginal acrylic cylinder was used once daily for 30 min continuously. The diameter and length of the cylinder were adapted to each participant’s vaginal dimensions measured at each follow-up visit. If the vaginal dimensions changed, the dilator was replaced by one of the appropriate sizes.

### Data collection

The women were evaluated at four different times: shortly after the end of radiotherapy, and four, eight, and 12 months after the beginning of the intervention. Clinical and sociodemographic data were obtained through an initial interview. Characteristics of the neoplasia and treatment were obtained from the medical records.

### Main outcome measure

#### Vaginal stenosis according to CTCAE v3.0 scale

Variation of vaginal canal amplitude and its possible interference with function was classified according to the Common Criteria for Adverse Events Version 3.0 (CTCAE v3.0) scale at the end of radiotherapy treatment, as well as four, eight, and 12 months after the beginning of the intervention. Grade 0: absence of vaginal stenosis; Grade 1: vaginal narrowing and/or shortening not interfering with function (absence of dyspareunia that would interfere with sexual intercourse); Grade 2: vaginal narrowing and/or shortening interfering with function (dyspareunia interfering with sexual intercourse); Grade 3: total occlusion of the vaginal canal that cannot be surgically corrected [6]. The evaluation of the CTCAE v3.0 scale was always performed by the same physician who specialized in radiotherapy.

#### Vaginal stenosis measured by percentage change in vaginal volume

Vaginal stenosis was measured by the percentage difference between the volume of the vagina soon after the end of radiotherapy treatment, and four, eight, and 12 months from the beginning of the intervention. The diameter and length of the vagina were measured using graduated cylinders, (25/30/35/40) mm in diameter and 1–20 cm in length. The physician estimated the diameter of the vaginal cylinder through vaginal digital evaluation. Then, a cylinder with the previously estimated diameter was inserted, coated with a lubricated condom to avoid discomfort (the cylinder should completely fill the vaginal canal, without leaving space and without causing discomfort to the patient). If the physician verified that the cylinder had not been perfectly adapted to the vaginal canal, the device was changed to better estimate the measurement. Then, the vaginal length was measured when the resistance of the vaginal bottom was felt at the end of the insertion of the device. This procedure was performed gently, effortlessly, to avoid muscle tension in the vaginal wall. The measurement was always performed by the same physician who specialized in radiotherapy. Vaginal volume was calculated in cubic centimeters using the formula below:

$$ \boldsymbol{V}=\frac{\mathbf{1}}{\mathbf{4}}\boldsymbol{\pi} {\boldsymbol{D}}^{\mathbf{2}}{\boldsymbol{L}}^{\ast}-\frac{\mathbf{1}}{\mathbf{2}\mathbf{4}}\boldsymbol{\pi} {\boldsymbol{D}}^{\mathbf{3}} $$

- ***V*** *= Vaginal volume*
- ***D*** *= Vaginal diameter*
- L* *= Vaginal length*

### Control variables

The control variables were: age; family income; body mass index (BMI, kg/m^2^); cylinder extension (cm); total dose of brachytherapy; race; marital status; menopausal state; number of vaginal deliveries; radiotherapy type; clinical tumor staging; vaginal wall invasion; tumor size; type of applicator; chemotherapy; type of radiotherapy; total duration of radiotherapy; smoking; surgery prior to radiotherapy; ovaries preserved before radiotherapy; sexual intercourse in at least one of the four follow-up evaluations.

### Sample size

The sample size was calculated based on the percentage of stenosis found in previous studies on estrogen, dilator, and lubricating gel treatments. Since testosterone had not yet been evaluated in studies for the prevention and treatment of vaginal stenosis, we chose to keep the number of women in the testosterone group equal to that of the vaginal dilator group. With that, we evaluated the result of its effectiveness as a topical hormonal intervention compared to the dilator, which is a mechanical intervention. For estrogen, the prevalence of stenosis was 56.8% in treated women and 79.6% in placebo users. Considering a test power of 80% and a significance level of 5%, the sample size was estimated at 65 cases [28]. For the dilator, the prevalence was 11% in treated women and 57% in placebo users. Considering the same test power and significance level, the sample size was estimated at 32 cases [29]. The sample was increased by 30% in each group to meet probable follow-up losses; the number of participants to be included in each group was 85 for the estrogen and lubricating gel groups and 42 for the vaginal dilator and topical testosterone groups, totaling 254 women invited to participate.

### Randomization

Cases were randomized based on the date, time, minutes, and seconds of randomization. The probability distribution was assumed to be multinomial, with p_1_ = 33.5% for the lubricating gel, p_2_ = 16.5% for the vaginal dilator group, p_3_ = 33.5% for the estrogen group and p_4_ = 16.5% for the testosterone group (where p_i_ = probability of occurrence of the i_th_ group). The list containing the numbers corresponding to randomization was held by only one of those responsible for the research. For each new case included in the study, a number was assigned corresponding to the intervention to be performed, which was only revealed after the end of the radiotherapy sessions, which corresponded to the beginning of the intervention period. SAS Software for Windows Version 9.2 (SAS Institute Inc., Cary, NC, USA) was used.

### Statistical analysis

To describe the sample profile, absolute and percentage frequency tables of categorical variables were made, and descriptive analysis of numerical variables was carried out using mean, standard deviation, median, minimum, maximum, and quartile values. To compare categorical variables between the four groups at baseline, the chi-square or Fisher’s exact tests were used. To compare the numerical variables between the four groups at the beginning of the study, the Kruskal-Wallis test was used due to absence of normal distribution of the variables. To assess a possible correlation between the two main outcome measures (CTCAE v3.0 scale and vaginal volume) before and after the intervention period, Spearman correlation tests were performed, both in the total sample and divided by treatment groups.

Statistical analysis was performed by intention to treat. The symmetry test was used to compare the incidence of vaginal stenosis between the four groups measured on the CTCAE v3.0 scale in the four evaluations during the intervention. To compare the percentage difference in vaginal volume between the four study groups after the intervention, the Kruskal-Wallis test was used. Subsequently, to assess factors independently associated with worsening vaginal stenosis on the CTCAE v3.0 scale, a multivariate Poisson regression model was constructed with stepwise variable selection criteria. Finally, to evaluate factors independently associated with the percentage decrease in vaginal volume, a multivariate linear regression model was built with stepwise variable selection criteria. The significance level was set at *p* < 0.05. For statistical analysis we used SAS for Windows Version 9.2.

## Results

**Fig. 1:**
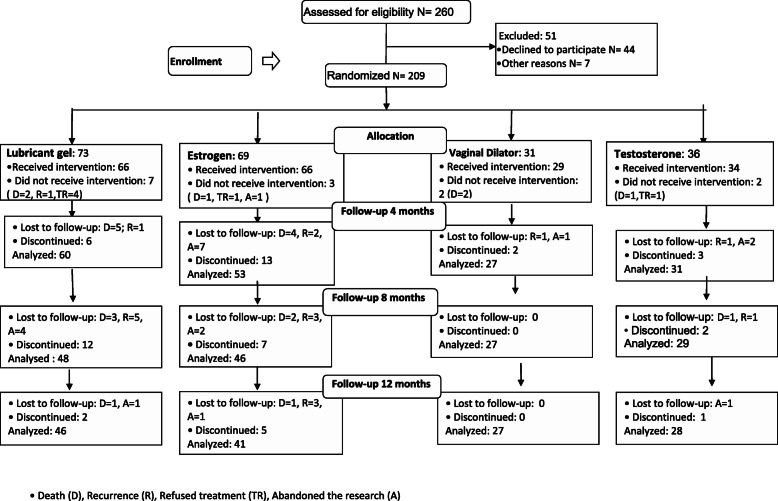
Study population between 2013 and 2018

From January 2013 to April 2017, 260 women who met the inclusion criteria were invited to participate in the study. Forty-four declined to participate and seven were not included due to changes in staging after further examinations. At the end of the inclusion period, 209 women were randomized: 69 to the estrogen group, 31 to the vaginal dilator group, 36 to the testosterone group, and 73 to the lubricating gel group. After the end of radiotherapy treatment, 66, 29, 34, and 66 women in the estrogen, dilator, testosterone, and lubricating gel groups, respectively started the intervention. After 12 months of follow-up, 41,27, 28, and 46 women in the estrogen, dilator, testosterone, and lubricating gel groups completed the evaluations. The flowchart of the study participants, containing the details of group discontinuations in the four evaluation periods, is shown in Fig. 1.

The mean age of the 195 women who started the intervention was 46.78 (± 13.01) years. Most of the women were Caucasian (61.54%), premenopausal (61.03%), had a partner (57.43%), and had not maintained sexual activity for the 3 months prior to radiotherapy (68.21%). Most women (73.84%) had advanced tumors (IIB–IIIB). The mean pelvic EBRT dose was 45.29 (± 2.43) Gy, 14.27 (± 0.68) Gy in the parametrium, and the mean brachytherapy dose was 26.65 (± 3.22) Gy. Most women (97.78%) received up to 45 Gy in the pelvis and a brachytherapy dose > 16 Gy (92.75%).

After randomization, the study intervention groups were homogeneous in the main control variables. In the group randomized to use lubricating gel, there was a higher proportion of women with tumors of dimensions ≤3 cm (*p* = 0.04) and with initial staging (*p* = 0.04); however, there were no significant differences regarding the extent of tumoral invasion into the vagina (*p* = 0.33). Comparisons between the main clinical and sociodemographic variables in the different clinical trial groups are shown in Table 1.

**Table 1:** Clinical and sociodemographic variables in the different clinical trial groups after randomization (*n* = 195)

	Testosterone ***n*** = 36	Estrogen ***n*** = 69	Vaginal dilator ***n*** = 31	Lubricant gel ***n*** = 73	***p***-value
**Age (mean)**	44.35 (±12.61)	45.88 (±12.61)	47.79 (±10.22)	48.50 (±14.60)	0.403^c^
**BMI (mean)**	28.23 (±5.68)	27.78 (±6.37)	27.43 (±5.43)	28.09 (±5.08)	0.791^c^
**Sexual intercourse per week (n)**					0.062^a^
0	16 (47.06%)	51 (77.27%)	20 (68.97%)	46 (69.70%)	
1	7 (20.59%)	7 (10.61%)	5 (17.24%)	12 (18.18%)	
≥ 2	11 (32.35%)	8 (12.12%)	4 (13.79%)	8 (12.12%)	
**Cylinder extension (cm)**					0.378^a^
≤ 3 cm	23 (67.65%)	38 (57.58%)	15 (48.39%)	32 (50.00%)	
> 3 cm	11 (32.35%)	28 (42.42%)	14 (48.28%)	32 (50.00%)	
**Race**					0.333^a^
White	21 (61.76%)	45 (68.18%)	14 (48.28%)	40 (60.61%)	
Non-white	13 (38.24%)	21 (31.82%)	15 (51.72%)	26 (39.39%)	
**Marital status**					0.440^a^
With partner	23 (67.65%)	37 (56.06%)	18 (62.07%)	34 (51.52%)	
Without partner	11 (32.35%)	29 (43.94%)	11 (37.93%)	32 (48.48%)	
**Menopausal status**					0.202^a^
Premenopausal	24 (70.59%)	41 (62.12%)	20 (68.97%)	34 (51.52%)	
Postmenopausal	10 (29.41%)	25 (37.88%)	09 (31.03%)	32 (48.48%)	
**Vaginal deliveries (n)**					0.803^a^
0	09 (26.47%)	15 (22.73%)	05 (17.24%)	13 (19.70%)	
≥ 1	25 (73.53%)	51 (77.27%)	24 (82.76%)	53 (80.30%)	
**Vaginal stenosis after radiotherapy (CTCAE v3.0)**					0.627^a^
0	11 (32.35%)	18 (27.27%)	04 (13.79%)	18 (27.27%)	
1	20 (58.82%)	44 (66.67%)	21 (72.41%)	43 (65.15%)	
2	03 (8.82%)	04 (6.06%)	04 (13.79%)	05 (7.58%)	
**Type of radiotherapy**					0.395^b^
EBRT	00 (0.00%)	00 (0.00%)	0 (0.00%)	02 (3.03%)	
Brachytherapy	01 (2.94%)	04 (6.06%)	04 (13.79%)	06 (9.09%)	
Both	33 (97.06%)	62 (93.94%)	25 (86.21%)	58 (87.88%)	
**Clinical stage**					**0.047**^**a**^
IB1- IIA	08 (23.53%)	11 (16.67%)	07 (24.14%)	25 (37.88%)	
IIB- IIIB	26 (76.47%)	55 (83.33%)	22 (75.86%)	48 (62.12%)	
**Ovaries preserved before radiotherapy**					0.649^b^
Yes	02 (5.88%)	09 (13.64%)	02 (6.90%)	07 (10.61%)	
No	32 (94.12%)	57 (86.36%)	27 (93.10%)	59 (89.39%)	
**Tumoral invasion of the vaginal walls**					0.339^a^
None	21 (61.76%)	36 (54.55%)	18 (62.07%)	37 (56.06%)	
Upper third	3 (8.82%)	19 (28.79%)	03 (10.34%)	14 (21.21%)	
Medium third	7 (20.59%)	08 (12.12%)	05 (17.24%)	8 (12.12%)	
Distal third	3 (8.82%)	03 (4.55%)	03 (10.34%)	7 (10.61%)	
**Tumor size (cm)**					**0.041**^**a**^
≤ 3 cm	11 (32.35%)	17 (25.76%)	7 (24.14%)	31 (46.97%)	
> 3 cm	23 (67.65%)	49 (74.24%)	22 (75.86%)	35 (53.03%)	
**Surgery**					0.409^a^
Yes	03 (8.82%)	10 (15.15%)	06 (20.69%)	14 (21.21%)	
No	31 (91.18%)	56 (84.85%)	23 (79.31%)	52 (78.79%)	
**Type of applicator**					0.365^a^
Cylinder	11 (32.35%)	27 (41.54%)	14 (48.28%)	32 (50.00%)	
Ring	23 (67.65%)	38 (58.46%)	15 (51.72%)	32 (50.00%)	
**Chemotherapy**					0.073^a^
Yes	27 (79.41%)	59 (89.39%)	22 (75.86%)	47 (71.21%)	
No	07 (20.59%)	07 (10.61%)	07 (24.14%)	19 (28.79%)	

*BMI* Body mass index

*EBRT* external beam radiotherapy

*CTCAE v3.0* Common Criteria for Adverse Events Version 3.0 scale

^a^Chi-square test

^b^Fisher’s exact test

^**c**^Kruskal-Wallis test

After the end of radiotherapy treatment and before the beginning of the intervention period, 51 women (26.1%) had Grade 0, 128 (65.6%) Grade 1 and 16 Grade 2 vaginal stenosis (8.2%). After 12 months, at the end of the intervention period, six women (4.2%) had Grade 0, 94 (66.2%) Grade 1, and 42 (29.6%) Grade 2 vaginal stenosis. The mean vaginal volume of the total group of women before the beginning of the intervention period was 115.43 mL (± 29.95), and at the end of the intervention period (12 months) it was 88.71 mL (± 31.37) – a reduction of 25.47%. A volume reduction occurred in 131 women (92.3%), six (4.2%) were unchanged, and five (3.5%) had increased vaginal volume (Table 2). We performed Spearman’s correlation tests to verify the association between vaginal stenosis according to CTCAE v3.0 scale and the vaginal volume both before and after the intervention period. Before the beginning of the intervention, we did not observe a significant correlation between vaginal volume and stenosis according to CTCAE v3.0 scale, both in the total sample, and divided between treatment groups. At the end of the intervention, we verified an association between stenosis according to CTCAE v3.0 scale and vaginal volume only in the group of women who received topical estrogen (*p* = 0.02; *r* = − 0.35) (data shown in supplementary material).

**Table 2:** Change in vaginal volume in the different intervention groups during follow-up (*n* = 142)

	Testosterone	Estrogen	Dilator	Lubricant gel	Total
	n (%)	n (%)	n (%)	n (%)	
**Reduction**	27 (96.4)	39 (95.1)	21 (77.8)	44 (95.6)	131 (92.3)
**Stable**	0	2 (4.9)	3 (11.1)	1 (2.2)	6 (4.2)
**Increase**	1 (3.6)	0	3 (11.1)	1 (2.2)	5 (3.5)
**Total**	28 (100)	41 (100)	27 (100)	46 (100)	142

Fisher’s exact test: 0.056

**Fig. 2:**
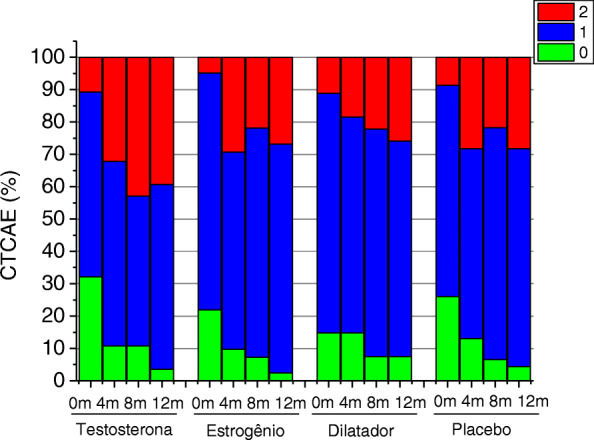
Vaginal stenosis (CTCAE v3.0 scale) in the different groups during follow-up (*n* = 142)

**Fig. 3:**
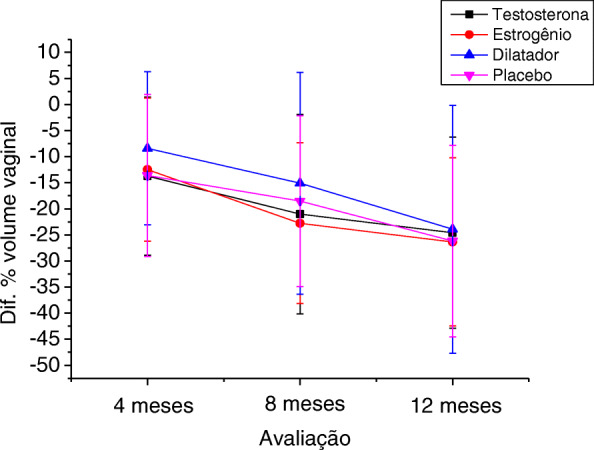
Change in vaginal volume (%) in the different groups during follow-up (*n* = 142)

By separately analyzing the evolution of vaginal stenosis in the different groups over the intervention period, significant worsening of the degree of stenosis (CTCAE v3.0 scale) was observed in the testosterone, estrogen, and lubricating gel groups. Women who used vaginal dilators did not have significant worsening (Table 3 and Fig. 2). When assessing the evolution of stenosis through the percentage change in vaginal volume over the intervention period, a similar reduction was observed between the four groups at all times evaluated, with no significant difference between the different types of treatment (Table 4 and Fig. 3).

**Table 3:** Vaginal stenosis (CTCAE v3.0 scale) after 12 months (*n* = 142)

Vaginal stenosis after radiotherapy	Vaginal stenosis after 12 months			Total	*p*-value*
	0	1	2		
**Testosterone**					***p*** **< 0.01**^**a**^
0	1 (3.57%)	**6 (21.43%)**	2 (7.14%)	**9 (32.14%)**	
1	0 (0.00%)	10 (35.71%)	**6 (21.43%)**	16 (57.14%)	
2	0 (0.00%)	0 (0.00%)	3 (10.71%)	3 (10.71%)	
**Total**	01 (3.57%)	16 (57.14%)	**11 (39.29%)**	28 (100.00%)	
**Estrogen**					***P*** **< 0.01**^**b**^
0	1 (2.44%)	**8 (19.51%)**	0 (0.00%)	**9 (21.95%)**	
1	0 (0.00%)	20 (48.78%)	**10 (24.39%)**	30 (73.17%)	
2	0 (0.00%)	1 (2.44%)	1 (2.44%)	2 (4.88%)	
**Total**	1 (2.44%)	29 (70.72%)	11 (26.83%)	41 (100.00%)	
**Vaginal dilator**					*p* = 0.372^c^
0	1 (3.70%)	2 (7.41%)	1 (3.70%)	4 (14.81%)	
1	1 (3.70%)	15 (55.56%)	4 (14.81%)	20 (74.07%)	
2	0 (0.00%)	1 (3.70%)	2 (7.41%)	3 (11.11%)	
**Total**	2 (7.41%)	18 (66.67%)	7 (25.93%)	27 (100.00%)	
**Lubricant gel**					***P*** **< 0.01**^**d**^
0	2 (4.35%)	**7 (15.22%)**	3 (6.52%)	**12 (26.09%)**	
1	0 (0.00%)	21 (45.65%)	**9 (19.57%)**	30 (65.22%)	
2	0 (0.00%)	3 (6.52%)	1 (2.17%)	4 (8.70%)	
**Total**	2 (4.35%)	31 (67.39%)	**13 (28.26%)**	46 (100.00%)	

*Symmetry test ^**a**^ S = 14.00; GL = 3; ^**b**^ S = 15.36; GL = 3; ^c^ S = 3.13; GL = 3; ^d^ S = 13.00; GL = 3

**Table 4:** Change in vaginal volume (%) in the different groups during follow-up (*n* = 142)

	N	Mean	SD	Min	Q1	Median	Q3	Max
**Testosterone**								
0-4^a^	31	−13.7	15.1	−43.1	−18.0	−10.6	0	10.7
4-8^b^	29	−21.0	19.1	−60.8	−40.5	−13.2	−7.5	10.7
8-12^c^	28	−24.6	18.3	−64.6	−41.8	−21.1	−8.8	10.7
**Estrogen**								
0-4^a^	53	−12.5	13.7	−51.4	−21.0	−9.6	0	10.6
4-8^b^	46	−22.8	15.4	−57.1	−36.4	−18.4	−9.7	0
8-12^c^	41	−26.3	16.1	−67.5	−37.0	−22.5	−13.8	0
**Dilator**								
0-4^a^	27	−8.4	14.65	−36.4	−21.4	−8.1	0	21.4
4-8^b^	27	−15.1	21.3	−49.7	−30.0	−13.5	0	36.0
8-12^c^	27	−23.9	23.8	−72.2	−39.2	−21.4	−5.7	12.0
**Lubricant gel**								
0-4^a^	60	−13.6	15.5	−68.5	−18.1	−9.7	0	9.7
4-8^b^	48	−18.5	16.4	−57.8	−32.2	−13.4	−6.3	10.7
8-12^c^	46	−26.2	18.4	−60.7	−45.1	−22.6	−10.7	10.7

Kruskal-Wallis test for comparison of mean values between the 4 groups

^a^0.622 ^b^0.407 ^c^0.844

To assess factors independently associated with worsening vaginal stenosis by the CTCAE v3.0 scale (including as independent variables the clinical trial groups), a multivariate Poisson regression model was constructed. The only variable independently associated with a worsening of the CTCAE scale was having reported sexual activity in at least one of the four evaluations performed during the intervention period (PR 2.27; 95% CI 1.15–4.50; *p* = 0.01) (data not shown). Finally, to evaluate the factors independently associated with the percentage decrease in vaginal volume (including the clinical trial groups as independent variables), a multivariate linear regression model was constructed. Performance of EBRT associated with brachytherapy (coefficient − 38.95; *p* < 0.01), not having had a vaginal delivery (coefficient − 20.31; *p* = 0.01), and having not reported sexual activity in any of the four evaluations during the intervention period (coefficient − 15.40; *p* = 0.02) were factors associated with a greater percentage reduction in vaginal volume.

## Discussion

Vaginal stenosis is a common adverse event following pelvic radiotherapy in women treated for cervical cancer. Apart from the impact on quality of life [30, 31], this may also hinder early diagnosis of tumor recurrences [9]. Therapy is unclear and guidelines are based on few clinical studies and experiences of individual cancer services [19].

The incidence of stenosis in the general group was high. At the beginning of the intervention period, 74% of women had some degree of stenosis assessed by the CTCAE scale. At the end of the study, considering only the 142 women who completed follow-up for 12 months, 96% had Grade I/II stenosis. These findings are different from those reported by Kirchheiner et al., who found a 59% crude incidence of vaginal stenosis (43% G1, 15% G2 and 1% G3) after a mean follow-up of 15 months [10]. We believe that this difference is due to the fact that Kirchheiner et al. used image-guided adaptive brachytherapy (IGABT) based on repeated volumetric imaging (computed tomography [CT], magnetic resonance imaging [MRI]), unlike our study, where 2D brachytherapy was used. It is known that one of the factors that influences the incidence of vaginal stenosis is high cumulative treatment dose [32]. In image-guided adaptive brachytherapy, it is possible to assess the exact dose administered at each treatment site. With this, it is possible to assess whether the dose limits of each structure are being respected, enabling the assessment of the percentage of vaginal volume receiving radiation. In the present study, women were submitted to 2D brachytherapy, that is, guided only by x-ray image with the anterior and lateral view of the treatment field. Thus, the doses in each structure were calculated at specific points, following the ICRU 38 [27] and it was not possible to calculate the dose received in areas other than these specific points. In a previous publication, we described a 1.74% reduction in diameter and 5.76% in mean length of the vagina in 139 women shortly after the end of radiotherapy for cervical cancer [33]. In the present study, using diameter and length measurements to estimate vaginal volume, we observed that the majority of women (92%) had a reduction in vaginal volume after 12 months of treatment, with a mean decrease of 25.47%.

Despite the rationalization that, by distending the vaginal walls, dilators may prevent adhesion formation in the mucosa and maintain vaginal patency [34], to date, no study has conclusively demonstrated the benefits of such use [20]. Through the CTCAE v3.0 scale, we observed that, among women who completed 12 months of follow-up, daily vaginal dilator use prevented the evolution (both onset and worsening) of vaginal stenosis. Daily vaginal dilator use appeared to favor the maintenance of sexual activity with less discomfort among these women, preventing the progression from Grade I to Grade II stenosis. However, daily use of a vaginal dilator did not prevent the decrease in mean vaginal volume, which occurred similarly among the four study groups. We emphasize the fact that six women randomized to vaginal dilator use had an increase in or maintenance of vaginal volume, with a trend of not reducing vaginal volume compared to other treatment groups (*p* = 0.056). Besides that, studies show that adherence to treatment with vaginal dilators may be small [35]. In our study compliance was assessed at follow-up visits by asking the patient if she was able to use the prescribed intervention without difficulty. If problems were identified, a new orientation was performed. Between the follow-up visits, if there were any doubts, women could contact the researchers for clarification. Even so, we observed that two women did not use the dilator correctly during the follow-up period. It is possible that, with greater adherence to treatment, significant benefit with respect to vaginal canal measurements may be achieved.

The use of vaginal dilators is not always tolerated by women with cervical cancer who may use them less frequently than directed or not at all. Therefore, investigation of other methods to prevent vaginal stenosis is essential [19]. Estrogen, the main regulating hormone in vaginal physiology, acts mainly on alpha-type estrogen receptors, which have the highest density in the deepest two-thirds of the vaginal canal [36, 37]. Additionally, some authors suggest that estrogen receptors are present in sensory and autonomic neurons of the vagina and vulva [38]. Genitourinary menopause syndrome (GSM) refers to the set of vulvovaginal signs and symptoms resulting from hypoestrogenism, involving changes in the major/minor lips, clitoris, vestibule, vagina, urethra, and bladder [17]. Estrogen therapy promotes vaginal cell growth, cell maturation, lactobacillus recolonization, increases vaginal blood flow, decreases vaginal pH to premenopausal levels, improves thickness, vaginal elasticity, and sexual response [36, 39]. An alternative treatment, tested in research protocols, is topical androgen therapy. This may act on specific receptors in the vaginal canal, or on estrogen receptors following peripheral conversion by the aromatase enzyme [24]. Both estrogen and androgen-based topical therapy for the treatment/prevention of post-radiotherapy vaginal stenosis have been poorly investigated to date. In the current analysis, the use of both estrogen and androgen were unable to prevent the progression of vaginal stenosis as assessed by the CTCAE scale and vaginal volume measurement.

As the study groups were not homogeneous in staging and tumor size, with a smaller number of tumors > 3 cm in size and with advanced stages in the group randomized to the lubricating gel group, we chose to perform two multiple analysis models, one for the CTCAE scale (Poisson regression) and one for the variation of the percentage of vaginal volume (linear regression). The only variable independently associated with a worsening of the CTCAE scale was having sexual activity in at least one of the four evaluations during the intervention period. We highlight the fact that this association is not due to a negative effect of sexual intercourse on vaginal health. This negative association may be related to the presence of symptoms during sexual activity such as bleeding and dyspareunia, which would classify women as having Grade II stenosis. That is, sexually active women are more exposed to the risk of complaining of stenosis affecting vaginal function when compared to women who have not had sexual intercourse during the intervention period. We believe that the use of a vaginal dilator was not significant in this regression analysis due to the small sample size.

Treatment with EBRT and brachytherapy, not having vaginal deliveries, and not having sex were factors independently associated with the percentage reduction in vaginal volume. According to previous studies, the association of EBRT and brachytherapy, high dose rate brachytherapy, and high doses of radiation are associated with a higher incidence and greater severity of vaginal stenosis [40, 41]. Traditionally, sexual activity is recommended to prevent vaginal stenosis [9]. This activity may help to distend the vaginal walls, resulting in a smaller reduction in vaginal canal volume. An alternative hypothesis is that sexually active women are easier to examine gynecologically, facilitating the measurement of the vaginal canal, resulting in more reliable measures. Similarly, with women who had had vaginal deliveries and possibly already had larger vaginal canal dimensions, gynecological examination and vaginal measurement would be easier.

This study has several limitations. Women included in the study were heterogeneous as to the type of cancer treatment to which they had been submitted. The group randomized to receive lubricating gel had a larger number of early-stage, smaller tumors. However, we emphasize the fact that tumor extension to the vaginal canal was similar between the four treatment groups. Sample size was calculated considering the prevalence of vaginal stenosis after estrogen and vaginal dilator treatment in previous studies. During the recruitment and intervention period, some factors prevented us from reaching the previously stipulated sample size. These included the high refusal to participate in the study, which increased the time planned for its completion. With the number of subjects who completed the intervention period, we estimate that the power of our sample to assess worsening vaginal stenosis using the CTCAE v3.0 scale was 44.9% and percentage change in vaginal volume was 10.8%. For a power of 80%, we would need 63 women to complete the study in each group to assess the CTCAE scale and 508 women per group to assess vaginal volume, which would be impossible due to the deadline for the end of the research. The CTCAE scale was also used in women who did not have sex before the evaluations. Because this scale takes into account the influence of the adverse event on organ function (dyspareunia interfering with sexual intercourse), this factor may have influenced the results. However, the number of sexually inactive women was similar in the four groups at the four time points evaluated; thus, the frequency of vaginal stenosis assessed by the CTCAE scale could be similarly influenced in the four groups.

Despite the limitations, we believe that the results obtained are valid. We emphasize the fact that the same physician performed all evaluations, both initial and follow-up, eliminating the possibility of interobserver variation. As demonstrated in our results, the lack of significant correlation between the two forms of evaluating vaginal stenosis corroborates the fact that they can be used in a complementary way. The assessment of the percentage change in vaginal volume allowed an objective assessment of the decrease in vaginal volume. The use of the CTCAE scale added the influence on organ function to the classification of severity of the objectively observed stenosis, complementing the assessment by volume. In our clinical experience, decreasing vaginal diameter in women with a small size before treatment results in greater interference with sexual function compared to women with larger diameter. Thus, the same percentage variation in vaginal volume may result in greater or lesser severity of stenosis for women depending on vaginal diameter before radiotherapy begins, highlighting the importance of using a scale that includes sexual function.

## Conclusion

Our data add information on therapeutic modalities for an adverse event that undermines the quality of life of cervical cancer survivors. In conclusion, there was a reduction in vaginal volume in all treatment groups analyzed, with no significant difference between them. However, women who used vaginal dilators had a lower frequency and severity of vaginal stenosis assessed by the CTCAE scale after 1 year of treatment.

## Supplementary Information

**Additional file 1.** Spearman correlation tests for CTCAE v3.0 scale and vaginal volume

## Data Availability

The datasets generated during and/or analyzed during the current study are available from the corresponding author on reasonable request.

## Abbreviations

BMI: Body mass index
CTCAE v3.0: Common Terminology Criteria for Adverse Events scale
EBRT: External beam radiotherapy
FAPESP: The São Paulo Research Foundation
ICRU 38: International Commission on Radiation Units & Measurements 38
UNICAMP: University of Campinas
