# Correction to: Topical estrogen, testosterone, and vaginal dilator in the prevention of vaginal stenosis after radiotherapy in women with cervical cancer: a randomized clinical trial

**DOI:** 10.1186/s12885-021-08543-8

**Published:** 2021-07-15

**Authors:** Jumara Martins, Ana Francisca Vaz, Regina Celia Grion, Lúcia Costa-Paiva, Luiz Francisco Baccaro

**Affiliations:** 1 Radiotherapy Section, Woman’s Hospital - Caism/Unicamp, Campinas, SP Brazil; 2 Department of Gynecology, Faculty of Medical Sciences, State University of Campinas - UNICAMP, Rua Alexander Fleming, 101, Cidade Universitária Zeferino Vaz, Campinas, SP 13083-881 Brazil

**Correction to: BMC Cancer 21, 682 (2021)**

**https://doi.org/10.1186/s12885-021-08274-w**

Following publication of the original article [1], it was noted that due to a figure processing error during typesetting, part of the text in Figs. 2 and 3 was presented in Portuguese. The correct Figs. 2 and 3 have been included in this Correction and the original article has been corrected.

**Fig. 2:**
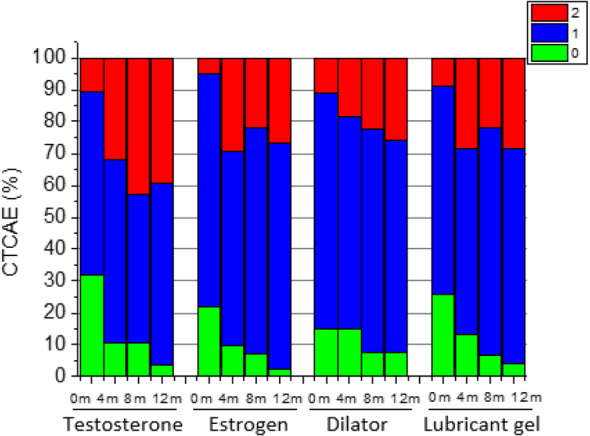
Vaginal stenosis (CTCAE v3.0 scale) in the different groups during follow-up (*n* = 142)

**Fig. 3:**
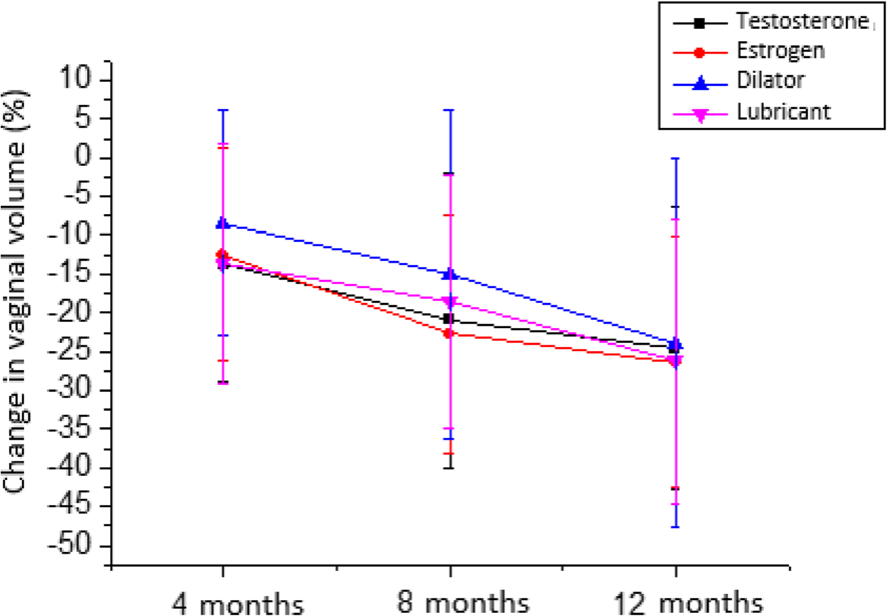
Change in vaginal volume (%) in the different groups during follow-up (*n* = 142)

The publisher apologises to the authors and readers for the inconvenience caused by the error.
