# Supplementary material for: Topical estrogen, testosterone, and vaginal dilator in the prevention of vaginal stenosis after radiotherapy in women with cervical cancer: a randomized clinical trial
Source: BMC Cancer. 2021 Jun 10;21:682. doi: 10.1186/s12885-021-08274-w (PMC8191143; doi:10.1186/s12885-021-08274-w)
Supplement: Supplementary file 1 — Additional file 1. Spearman correlation tests for CTCAE v3.0 scale and vaginal volume [file 12885_2021_8274_MOESM1_ESM.docx]

**Supplementary material**

Spearman correlation tests for CTCAE v3.0 scale and vaginal volume

| **CTCAE v3.0 scale** | **Vaginal volume before intervention** | **CTCAE v3.0 scale** | **Vaginal volume after intervention** |
| --- | --- | --- | --- |
| Total sample (n=195) |  | Total sample (n=142) |  |
| r | -0.11949 | r | -0.10373 |
| p | 0.0962 | p | 0.2193 |
|  |  |  |  |
| Testosterone (n=34) |  | Testosterone (n=28) |  |
| r | -0.27470 | r | -0.34943 |
| p | 0.1159 | p | 0.0684 |
|  |  |  |  |
| Estrogen (n=66) |  | Estrogen (n=41) |  |
| r | -0.14380 | r | -0.35643 |
| p | 0.2494 | p | 0.0222 |
|  |  |  |  |
| Dilator (n=29) |  | Dilator (n=27) |  |
| r | 0.04761 | r | 0.06769 |
| p | 0.8063 | p | 0.7373 |
|  |  |  |  |
| Lubricant (n=66) |  | Lubricant (n=46) |  |
| r | -0.06330 | r | 0.08308 |
| p | 0.6136 | p | 0.5830 |

* r = Spearman correlation coefficient; p = p-value; n = number of subjects
